# Perihematomal glutamate level is associated with the blood–brain barrier disruption in a rabbit model of intracerebral hemorrhage

**DOI:** 10.1186/2193-1801-2-358

**Published:** 2013-07-30

**Authors:** Guofeng Wu, Shujie Sun, Fei Sheng, Likun Wang, Fan Wang

**Affiliations:** Department of Neurology, Affiliated Hospital, Guiyang Medical College, No. 28, Guiyijie Road, Liuguangmen, Guiyang City, Guizhou Province 550004 P R China; Emergency Department of Affiliated East Hospital, Tongji University, Shanghai, 200120 P R China

**Keywords:** Intracerebral hemorrhage, Perihematomal glutamate, Brain water content, Blood–brain-barrier, Evans blue

## Abstract

**Objective:**

To observe the relationship between the perihematomal glutamate levels and the blood–brain barrier (BBB) permeability in a rabbit model of intracerebral hemorrhage (ICH).

**Methods:**

Seventy-two rabbits were randomly divided into an intracerebral hemorrhage (ICH) model group and a normal control (NC) group, and each group of 36 rabbits was subsequently divided into 6, 12, 18, 24, 48 and 72 h groups (n = 6 each). An ICH model was induced by stereotactic injection of autologous, arterial, non-anticoagulated blood into rabbit basal ganglia. The same procedures were performed in the NC group, but blood was not injected. The rabbits were sacrificed at specific time points after the experiment began depending on their group. Perihematomal brain tissues were collected to determine glutamate levels, BBB permeability and brain water content (BWC).

**Results:**

All of the assessed parameters were increased 6 hour after blood infusion and continued to gradually increase, peaking at 48 hours. Differences were observed when ICH values were compared with those of the NC group (*p <* 0.05).

**Conclusions:**

Perihematomal glutamate increased significantly after ICH. High levels of glutamate are closely associated with BBB disruption and the brain edema. Therefore, glutamate may play an important role in the pathogenesis of secondary brain injury after (ICH).

## Introduction

Spontaneous intracerebral hemorrhage (ICH) is a devastating neurological disorder with considerable mortality and morbidity that accounts for 20%-30% of acute cerebral vascular disease. However, no currently available intervention has been shown to alter the outcome of patients who have suffered acute ICH (Parker et al. 2010). Secondary brain damage, which often occurs in the days following the initial hemorrhage, is closely associated with significant neurological deterioration (Miller et al. 2007). ICH negatively affects BBB permeability, which results in neuronal dysfunction (Shi et al. 2011). A hallmark of ICH-induced brain injury, BBB disruption is accompanied by brain edema in the surrounding areas of ICH. The degree of BBB breakdown has been directly correlated with late functional outcome (Lampl et al. 2005). A range of factors including inflammatory mediators, thrombin and hemoglobin breakdown products are involved in BBB disruption (Keep et al. 2008).

Recently, some studies have observed a relationship between the perihematomal glutamate levels and the secondary brain injury in ICH models, and the impact of glutamate on ICH patient outcome (Miller et al. 2007; Chiang et al. 2006; Hartings et al. 2008; Qureshi et al. 2003; Wang et al. 2008a). Glutamate accumulates during the early period of experimental hematoma, and the activation of N-methyl-D-aspartate (NMDA) receptors by glutamate can result in an influx of calcium (Ca^2+^) and subsequent neuronal death (Qureshi et al. 2003; Lee et al. 2006). In cases of ICH, increased levels of glutamate and aspartate that correlate with neurological status have been detected after subarachnoid hemorrhage (SAH) (Germano et al. 2007), and glutamate-mediated excitotoxicity is a major consequence of stroke (Hazell 2007). Blockade of NMDA or AMPA receptors could attenuate BBB disruption in focal cerebral ischemia, and treatment with magnesium, MK-801 and a combination of magnesium and MK-801 can reduce brain edema formation and can help restore BBB integrity after experimental diffuse brain injury (Imer et al. 2009). Reducing perihematomal glutamate level by minimally invasive procedures for intracerebral hematoma also decreases BBB permeability and BWC (Wu et al. 2011). These results demonstrate that secondary brain damage is associated with glutamate-related excitotoxicity, and glutamate levels are closely associated with ICH patient outcome. However, the exact relationships among elevated glutamate levels in perihematomal brain tissues, BBB permeability and brain edema have not been thoroughly investigated.

The objective of the current study was to determine the relationship between the perifocal glutamate levels and the BBB permeability in a rabbit model of ICH.

## Methods and materials

### Materials

#### Main reagents

Formamide (molecular formula: HCONH_2_, Chongqing Chuanjiang Chemical Reagent Factory, Chongqing,China), urethane (molecular formula: C3H7NO2, Wuxi Yangshan Biochemical), Evans blue (Beijing Hengye Zhongyuan Chemical), 4% paraformaldehyde (Wuhan Boster Biological Technology), urokinase (Guangdong Livzon Pharmaceutical), glutamate (Sigma), derivatization reagent borate buffer (Agilent Technologies, USA), FMOC reagent Agilent PN5061-3337 (Agilent Technologies, USA), OPA reagent Agilent PN5061-3335 (Agilent Technologies, USA, 2,4-DNFB (Japan) and HPLC-grade acetonitrile and methanol (Germany) were used in this study.

#### Main instruments

We used the following instruments: a ZH-Lanxing B-Type rabbit stereotaxic Apparatus (Huaibei Zhenghua Biological Instrument & Equipment), electronic scales (Satourious, Germany), a Rainbow Type-722 grating spectrophotometer (Shandong Gaomi Rainbow Analytical Instrument), a 5415R high-speed centrifuge (Frozen, Heraeus Company), micropipettes (Eppendorf), a 202–2 constant temperature oven (Shanghai Luda Laboratory Apparatus), a digital display thermostat water bath (HH-2; Guohua Electric Appliance), a tabletop general centrifuge (TGL-16B; Shanghai Anting Scientific Instrument Factory), a −80°C freezer (Forman Scientific Company), a refrigerator (Qingdao), a CT provided by the Guiyang Medical College, a high-performance liquid chromatograph (HP-1100; Agilent Technologies, USA), a G1315 A diode-array detector (DAD, Agilent Technologies, USA), a pH meter (410 A, ORION, USA), an Agilent 1313A Automatic Sampler (Agilent Technologies, USA), a column oven (Agilent Technologies, USA) and scales (Beijing Gangdong Hengye Instrument).

#### Experimental groups

The present study was approved by the Animal Care and Use Committee of Guiyang Medical College.

Seventy-two male rabbits (2.8-3.4 kg) were provided by the Animal Center of Guiyang Medical College. The animals were divided randomly into an ICH group and a NC group (n = 36 each), and they were equally divided into 6 subgroups (n = 6 each) that were sacrificed at 6, 12, 18, 24, 48 or 72 h after ICH induction. ICH was induced in all animals in the model group.

### Animal preparation

#### ICH model preparation

Rabbits were fasted for 12 hours and water restricted for 4 hours prior to the experiment. The rabbits were then anesthetized by injecting 20% urethane (5 ml/kg) into the ear vein. Slow breathing, a slow corneal reflex and no pain reaction were used as indicators of complete anesthetization. The head of the rabbit was then shaved to expose the skin for surgery.

The anesthetized rabbit was fastened to the stereotaxic apparatus, and the skin in the operation field was disinfected with 75% alcohol. A 3-cm incision was made along the mid-line between the two post-orbital margins, and the subcutaneous fascia was stripped to expose the skull. A 3% H_2_O_2_ solution was used to open the periosteum and expose the bregma and lambdoid sutures. The head was then adjusted to make the bregma 1.5 mm higher than the lambdoid suture. The position of the internal capsule was located according to the rabbit stereotaxic atlas. The coronal plane crossing the center of bregma was used as the coronal zero plane (AP0); A1 represented the coronal plane 1 mm rostral to the AP0, and the internal capsule was estimated to be between A5 and P2. The present experiment used the A1 level and the bregma as base points, with the puncture point 6 mm left of the coronal suture and 1 mm parallel to the sagittal suture. A hole was drilled in the skull, and a #12 needle and a 1-ml syringe were used to deliver 0.8 ml autologous arterial blood taken from the central ear artery. The syringe was then connected to a #7 needle in without a tip. Air was completely removed from the syringe, leaving 0.3 ml of blood. The #7 needle was quickly inserted vertically 12 mm into the skull, and the blood was slowly injected into the basal ganglia. The injection lasted for approximately 3 minutes. The needle was left in place for 8 min after the injection to prevent blood backflow, and then the needle was slowly removed. Local hemostasis was induced by compression for 2 min.

The drill hole was then covered using gutta-percha. A CT scan was performed 3 hours later. A hyperdensity shadow in the basal ganglia region without a shadow in the lateral ventricle was considered as a successful model of ICH.

After successful ICH induction confirmed by CT scan, the animals were returned to the housing facility. All the animals recovered from anesthesia within 5 hours after intravenous injection of 20% urethane. Exclusion criteria included visualization of back flow along the needle track, blood in the ventricle, and death.

#### NC group treatment

Procedures performed in the ICH group were identical in the NC group, but autologous arterial blood was not injected into the basal ganglia to induce intracerebral hematoma.

#### Medical treatment of the animals

Animals received an intramuscular injection of penicillin (400,000 U) to prevent infection. They were housed as usual until they were sacrificed. No other medical treatment was administered.

### Intracerebral hematoma volume and neurological deficit score

To demonstrate that ICH was successfully modeled, hematoma volume was measured with a CT scan, and a neurological deficit scale was used to assess neurological functions (Purdy et al. 1989). The scale included tests of motor function (Parker et al. 2010; Miller et al. 2007; Shi et al. 2011; Lampl et al. 2005), consciousness (Parker et al. 2010; Miller et al. 2007; Shi et al. 2011; Lampl et al. 2005), head turning (0–1), circling (0–1) and hemianopsia (0–1). A total score of 11 indicates maximum impairment (comatose or dead rabbit), whereas 2 denotes complete normality. Tests were conducted by an observer blinded to the animals. The tests were conducted by an observer blinded to the animal’s group.

### Brain tissues preparation

A 2% Evans blue (2 ml/kg) solution was injected into the ear vein 2 hours before euthanization. The animals were then anesthetized using 20% urethane, and the chest was quickly opened to expose the heart. A tube was inserted from the left ventricle into the aortic root, with a small hole cut in the right ventricle to allow the tube to exit. Rabbits were transcardially perfused with 400 ml 0.9% sodium chloride solution until the fluid flowed clear, at which time they were perfused with 100 ml 4% paraformaldehyde. The brain was then extracted and placed on ice. The needle track was used as the center to prepare coronal and sagittal sections, then the brain on the hematoma side was cut and divided into four parts: front-inner, front-outside, back-inner and back-outside. A total of 5 mm of brain tissue surrounding the hematoma was collected from each area. The front-inner and front-outside parts were used for amino acid testing, the back-inner part was used for assessing Evans blue content, and the back-outside part was used for testing BWC.

### Perihematomal glutamate level measurement

Perihematomal glutamate content was determined using high-performance liquid chromatography.

#### Chromatographic conditions

A ZORBAX Eclipse-AAA (4.6 × 150 mm, 5 μm) chromatographic column was used. Mobile phase A was 40 mM Na_2_HPO_4_, pH 7.8 (5.5 g Na_2_HPO_4_ · H_2_O + 1 l water, NaOH was added to make the pH 7.8) and mobile phase B was 45:45:10 (V/V/V) ACN: MeOH:water. The column was run with a flow rate of 2 ml/min. Phase B increased from 0 to 57% between 0 and 18 min and from 57 to 100% between 18.1 and 18.6 min. It remained at 100% between 18.6 and 22.3 min and decreased from 100% to 0% between 22.3 min and 23.2 min. Between 23.2 and 26 min, Phase B remained at 0%. The column temperature was 40°C, and the sampling volume was 10 μl. The diode array wavelength was 262 nm, and the reference wavelength was 324 nm.

#### Derivative solution preparation

A total of 25 mg OPA was dissolved in 1 ml methanol. Sodium borohydride buffer (4 mol/l) was then added (pH 10.4), and the solution was stirred. The final solution was stored at 4°C.

#### Standard solution preparation

Glutamate and 0.2 mol/l NaHCO_3_ (pH 9.8) was used to make a 1-g/l standard stock solution.

#### Biosample preparation

The brain tissue was defrosted, weighed and placed in a dry glass homogenizer. Dilute hydrochloric acid (1:5 w/v, 0.1 mmol/l) was added, and the homogenized brains were placed into an ultrasonicator (Temp: 4°C; pulse for 2 s, rest for 2 s; intensity: 20%; 15 times in total). Samples were then centrifuged at 1,200 rpm for 20 min at 4°C. Borate saline buffer (2.5 μl) was then added to the supernatant solution and mixed for 20 min, followed by the addition of 0.5 μl OPA. The sample was then mixed for 30 s before FMOC (0.5 μl) was added and the solution was mixed for another 30 s. Finally, water (32 μl) was added, and the final sample was mixed for 30 s, and 10 μl of the sample was used.

#### Calculation of glutamate concentration

The peak area of glutamate from the HPLC was integrated and used as an external standard for the samples. The glutamate concentration for 1 g of brain tissue was then calculated according to the sample quality.

### Determination of BBB permeability

#### Experimental methods

Evans blue was used as a tracer to measure BBB permeability. Two hours before each experiment, 2% Evans blue (2 ml/kg) was injected into the ear vein. After 2 h, the brain tissue was quickly removed. The tissue surrounding the hematoma was weighed (within 0.1 mg) and then placed into a test tube with 4 ml formamide. The tube was then capped and placed in a 54°C water bath for 24 h to allow the Evans blue to spread throughout the brain tissue.

The samples were then centrifuged at 2,400 rpm for 5 min. The supernatant was placed in a quartz cuvette, and a spectrophotometer was used (λ = 632 nm) to measure the absorbance of the supernatant. Formamide was used as a blank control.

#### Setting up the standard curve

Evans blue (4 mg) was placed into a volumetric flask and weighed (within 0.1 mg). A total of 100 ml NS was added, and the solution was stirred. From this solution, 0.3 ml was removed and placed in 5.7 ml of formamide to make the standard buffer solution. A total of 3 ml of this solution was serially diluted in seven tubes each containing 3 ml formamide. The amount of Evans blue in each of the seven tubes was 8, 4, 2, 1, 0.5, 0.25 and 0.125 μg/ml. The tubes were capped and placed into a 54°C water bath for 24 hours. The aforementioned method was used to measure absorbance. Linear regressions were calculated for the absorbencies and Evans blue content. The final equation was y = 0.0053 × + 0.0608 (R2 = 0.9833).

#### Evans blue content computation

We used the formamide method to measure Evans blue content in brain tissue to assess BBB damage severity. The formula used was as follows: Evans blue content in brain tissue (μg/g wet brain) = B × formamide (ml)/wet weight (g), where B refers to the Evans blue content of the sample (μg/ml) given by the linear regression equation according to standard curve.

### Measurement of BWC

The dry and wet weight method was used to measure BWC. The brains were quickly removed, and brain tissue from the back-outside portion of the hematomas was used. First, the weight of the wet tissue was obtained. The samples were then placed in an oven at 100°C for 48 h, and the dried samples were then weighed. BWC was then calculated as (wet weight–dry weight)/wet weight × 100%.

### Statistical analysis

All data were analyzed using SPSS 11.5. Basic data are expressed as the mean ± standard deviation (X ± SD). A Kruskal-Wallis test was used to make comparisons across the whole time series among the groups. If the Kruskal-Wallis test detected significant differences, Bonferroni corrections were used to make comparisons between groups. A correlative analysis was also performed to assess the relationship between the glutamate level and the BBB permeability. A *p* value less than 0.05 was considered statistically significant. Statistical analysis was performed with the help of the Department of Biostatistics of Guiyang Medical College.

## Results

**Figure 1:**
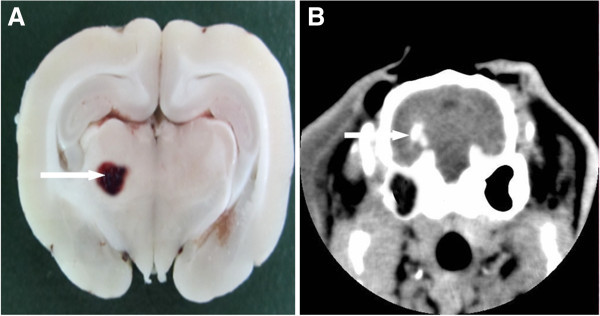
**Brain CT and histological section showing hematoma in the rabbit`s brain.** The arrow pointed to the basal ganglia hematoma on histological section **(A)** or brain CT **(B)**.

Following blood infusion into the basal ganglia, the animals were unable to stand up or crawl. The contralateral extremities were less responsive to noxious stimulation. Brain CT showed an oval or round hyperdensity in the basal ganglia (Figure 1). The abovementioned observations demonstrate that the ICH model in this study was successful and reliable.

Three rabbits were excluded from this study, one animal died of overdose of anesthetic agents during surgery, and the other two did not show evidence of hematoma formation. A total of 33 rabbits in the ICH group (5 in the 6 h group, 6 in the 12 h group, 6 in the 18 h group, 5 in the 24 h group, 5 in the 48 h group and 6 in 72 h group) and 36 rabbits in the NC group were included in the present study.

### Hematoma volume and neurological function

The hematoma volume and neurological deficit score in each subgroup were determined after the model was successfully induced. The average hematoma volume ranged from 0.250 ± 0.014 ml to 0.266 ± 0.012 ml, and no significant difference was observed among subgroups. The neurological deficit score (8.833 ± 0.753 to 9.167 ± 0.817) increased immediately after the blood was infused in the ICH group compared with the NC group, and no remarkable difference was noted among subgroups. These results suggested that the ICH model was successful.

### Perihematomal glutamate level

The glutamate level began to increase 6 hours after blood infusion. It gradually increased over time and peaked at 48 hours, which was significantly different compared with the NC group *(p* < 0.05). The glutamate level changed over time after blood was infused into the brain (*χ*^2^ = 20.134). When comparisons were performed between subgroups, the glutamate level was lowest in 6-hour group, also the Evans blue (*χ*^2^ = 20.134) and the BWC (*χ*^2^ = 20.142). These values were highest in 48-h group (Wilcoxon W = 27 when the 6-h group was compared with the 48-h group, Wilcoxon W = 21 when the 6-h group was compared with the other groups), suggesting that less brain damage had occurred in the early stage after ICH, indicating that it might be an optimal time window for intervention (Table 1).

**Table 1:** **Changes of perifocal glutamate level, Evan`s blue and BEC in a rabbit model of ICH**

Time point	Glutamate content(μg/g)		Evan`s blue content(μg/g)		Brain water content(%)	
	ICH group	NC group	ICH group	NC group	ICH group	NC group
6 hour	2286.34 ± 43.78◇#	2219.11 ± 33.67	28.86 ± 0.87◇#	4.14 ± 0.21	78.14 ± 0.74◇#	77.17 ± 0.44
12 hour	2317.64 ± 65.18	2184.51 ± 47.12	29.19 ± 1.06	3.96 ± 0.13	79.86 ± 0.54	77.06 ± 0.43
18 hour	2361.54 ± 51.84	2231.32 ± 39.54	32.19 ± 0.74	4.21 ± 0.25	81.19 ± 0.41	76.98 ± 0.81
24 hour	2438.62 ± 42.56	2213.98 ± 50.38	33.76 ± 1.18	4.17 ± 0.18	81.65 ± 0.82	77.45 ± 0.91
48 hour	2479.13 ± 59.67	2198.36 ± 42.87	35.36 ± 1.18	4.08 ± 0.27	81.92 ± 0.68	77.38 ± 0.67
72 hour	2480.23 ± 49.60	2210.88 ± 45.66	34.96 ± 1.20	4.20 ± 0.22	81.96 ± 0.53	77.43 ± 0.78

◇Compared with the NC group (P < 0.05). #Compared with other subgroups (P < 0.05).

In the NC group, no significant differences were observed between subgroups at different time points (*p* > 0.05), suggesting that the simple puncture of brain tissue with a needle has little impact on glutamate levels in brain tissue around the needle tract (Table 1).

### BBB permeability and BWC

Evans blue and BWC around the hematoma began to increase 6 hours after successful ICH induction, increased gradually over time after blood infusion and peaked at 48 hours (Wilcoxon W = 27 when the 6 h group was compared with 48 h group, Wilcoxon W = 21 as 6 h group was compared with the other groups) ,suggesting that BBB disruption began in the early stage. Compared with the NC group, significant differences were observed for Evans blue content at different time points (at 6 h, 12 h, 18 h, 24 h, 48 h and 72 h) ,also the brain water content The same phenomenon was observed in perihematomal brain water content.

These results suggest that BBB permeability and brain edema both increased (Table 1). The range of the brain edema was observed on histological sections. Tissue approximately 5 mm around the hematoma became edematous, but the results were not quantified.

No significant difference was observed in Evans blue content or BWC among subgroups at different time points (*p* > 0.05) in the NC group, suggesting that a simple puncture of brain tissues does not decrease BBB permeability around the needle tract.

### Correlation between glutamate level and BBB disruption

**Figure 2:**
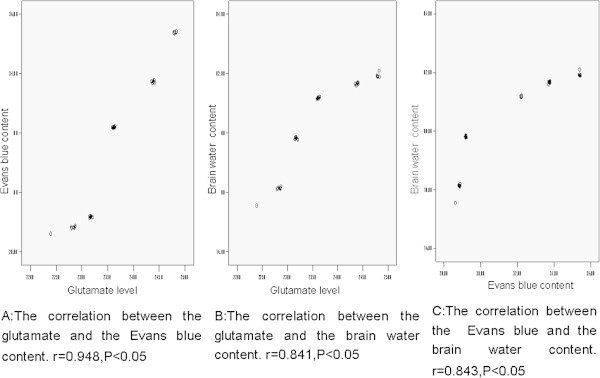
**Correlation between the glutamate level and BBB disruption and brain edema formation.** The perihematomal glutamate content has a positive correlation with the Evans blue content **(A)** and the BWC **(B)**. The same correlation was also observed between the the Evans blue content and the BWC **(C)**.

A correlation analysis was performed to analyze the relationship between perifocal glutamate levels and BBB permeability. Positive correlations was observed between elevated glutamate levels and BBB permeability (r = 0.948, *p* < 0.05) and between glutamate level and BWC (r = 0.841, *p* < 0.05). The same correlation was also observed between BBB permeability and BWC (r = 0.841, *p* < 0.05). We found that the higher glutamate levels were associated with more serious BBB disruption and cerebral edema, suggesting that the perihematomal glutamate level was highly associated with BBB disruption and brain edema formation (Figure 2). Further observation demonstrated that the glutamate vs. Evans blue and glutamate vs. BWC plots display a sigmoidal relationship.

## Discussion

Intracerebral hemorrhage remains the least treatable form of stroke (Rincon & Mayer 2008). ICH models serve as an important tool for understanding mechanisms underlying brain injury after an intracerebral bleed (Andaluz et al. 2002). Based on previously published studies of an ICH model (Andaluz et al. 2002; Frantzias et al. 2011), we successfully generated a rabbit model of ICH by stereotactic injection of 0.3 ml non-anticoagulant autologous arterial blood into the basal ganglia. The volume of the rabbit`s brain is approximately 1 percent of the human brain (Narushima et al. 2003), so this injection is approximately equivalent to about 30 ml of hematoma in the human brain. In the present study, an ICH model was induced successfully in 33 rabbits out of 36 ones. Three rabbits were excluded from the study as there is no evidence of hematoma formation. For experimental purposes, the most common technique of producing an intracerebral hematoma in rabbits is the injection of unclotted autologous blood. Sometimes,the injected blood may ruptures into the ventricular system or it extends to the subarachnoid or subdural space (Deinsberger et al. 1996). Therefore, a CT scan was performed after successful ICH injection 3 hours later. If postoperative brain CT does not show an oval or round hyperdensity in the basal ganglia,we do not consider that there is a hematoma formation.

ICH is always followed by blood–brain barrier disruption, which contributes to the enhanced BBB permeability and vasogenic brain edema in the surrounding areas. Brain edema is one of the most frequent and serious complications of ICH, but how the ICH causes brain edema remains unknown (Wu et al. 2008). Previously published reports demonstrated that a range of factors are involved in BBB disruption (Keep et al. 2008). Studies in recent years have observed a relationship between perihematomal brain tissue glutamate levels and secondary brain injury in models of ICH and the impact of glutamate on ICH patient outcome (Miller et al. 2007; Chiang et al. 2006; Hartings et al. 2008; Qureshi et al. 2003; Wang et al. 2008a). Glutamate is accumulated during the acute period of experimental hematoma, and the activation of NMDA receptors by glutamate can result in calcium influx and neuronal death following ICH (Lee et al. 2006). Secondary brain damages are associated with glutamate-related excitotoxicity, and the level of glutamate is closely associated with ICH patient outcome. Patients with spontaneous ICH who presented with more serious brain injuries had a higher concentration of glutamate in the brain and poorer prognosis (Wang et al. 2008b).

In the present study, perifocal glutamate levels began to increase 6 hours after infusing blood into the basal ganglia. As the time after infusing the blood into the brain was prolonged, the glutamate level increased gradually and peaked 48 hours after successful ICH induction. The perifocal glutamate level varied in subgroups at different time points. It was lowest in the 6-h group and highest in the 48 and 72 h group. We observed statistically significant increases of perihematomal glutamate at different time points compared with the NC group, suggesting that glutamate level increase is a pathological process that follows ICH.

Evans blue is a type of dark blue powdered dye that is unable to penetrate the BBB in normal conditions. The Evans blue assay is a popular method for quantifying BBB disruption. A small amount of Evans blue was observed in normal animal brains after intravenous injection (Wu et al. 2011). In the present studies, perihematomal Evans blue content was significantly increased in the ICH groups at different time points compared with the control group, demonstrating that the BBB was disrupted after the intracerebral infusion of blood. BBB disruption was followed by brain edema formation. Perihematomal BWC began to increase in the 6-h group and peaked 48 hours after ICH was induced. The change of BWC was consistent with enhanced BBB permeability. The range of edematous brain area was observed on histological sections. An area extending at least 5 mm from the hematoma became edematous, but because we only collected a total of 5 mm of brain tissue around the area to measure BWC, we were unable to precisely quantify the range of brain edema. This is a limitation of the present study.

The increased perifocal glutamate positively correlated with enhanced BBB permeability and increased BWC. Higher perihematomal glutamate levels were indicative of more serious BBB disruption and brain edema. Further observation demonstrated that the glutamate vs. Evans blue and glutamate vs. BWC plots display a sigmoidal relationship.,suggesting that the BBB permeability or the BWC increased slightly in the early stage as the glutamate level elevated. They increased dramatically as the glutamate level continue to increase. However, the increase of the BBB permeability or the WBC become lower after the glutamate level reaches to a certain extent. The relationship between the Evans blue and the BWC also display similar results.

However, the mechanism underlying increased perihematomal glutamate remains poorly understood. One possibility is an influx of glutamate from the initial hematoma into the extracellular space during ICH (Wagner et al. 1998). It is also possible that glutamate enters perihematomal brain tissue from the bloodstream following BBB disruption. Although the mechanism behind increased glutamate after ICH remains poorly understood, glutamate has been demonstrated to be involved in BBB disruption (Fu et al. 2007). It is reasonable for us to postulate that perihematomal BBB disruption is due to increased glutamate content. High levels of the glutamate activate NMDA receptors, consequently resulting in the entry of large amount of Ca^2+^ into the neurons, resulting in neuronal necrosis or apoptosis (Nicholls et al. 2007). Elevated glutamate levels in perihematomal brain tissues resulted in increase BBB disruption and permeability, thereby aggravating brain edema. Our results corroborate these findings. Because perihematomal glutamate level is closely associated with BBB disruption and brain edema formation, reducing glutamate levels or antagonizing its effect in the early stages of ICH might be effective in treating secondary brain edema. However, the current study focused on the relationship of glutamate levels and the BBB permeability, and because the animals were sacrificed 72 hours after ICH induction, we were unable to observe a correlation between glutamate levels and neurofunctional recovery.
